# ISG15 and ISGylation modulates cancer stem cell-like characteristics in promoting tumor growth of anaplastic thyroid carcinoma

**DOI:** 10.1186/s13046-023-02751-9

**Published:** 2023-07-27

**Authors:** Tong Xu, Chaozhuang Zhu, Jinming Chen, Feifeng Song, Xinxin Ren, Shanshan Wang, Xiaofen Yi, Yiwen Zhang, Wanli Zhang, Qing Hu, Hui Qin, Yujia Liu, Song Zhang, Zhuo Tan, Zongfu Pan, Ping Huang, Minghua Ge

**Affiliations:** 1 Center for Clinical Pharmacy, Cancer Center, Department of Pharmacy, Zhejiang Provincial People’s Hospital (Affiliated People’s Hospital), Hangzhou Medical College, Hangzhou, Zhejiang China; 2 Zhejiang University of Technology, Hangzhou, Zhejiang China; 3 Hangzhou Medical College, Hangzhou, Zhejiang China; 4 Key Laboratory of Endocrine Gland Diseases of Zhejiang Province, Zhejiang Provincial People’s Hospital (Affiliated People’s Hospital), Hangzhou Medical College, Hangzhou, Zhejiang China; 5 Otolaryngology & Head and Neck Center, Cancer Center, Department of Head and Neck Surgery, Affiliated People’s Hospital, Zhejiang Provincial People’s Hospital, Hangzhou Medical College, Zhejiang Hangzhou, China

**Keywords:** ISG15, ISGylation, Anaplastic thyroid carcinoma, Cancer stem cell-like characteristics

## Abstract

**Background:**

Anaplastic thyroid carcinoma (ATC) was a rare and extremely malignant endocrine cancer with the distinct hallmark of high proportion of cancer stem cell-like characteristics. Therapies aiming to cancer stem-like cells (CSCs) were emerging as a new direction in cancer treatment, but targeting ATC CSCs remained challenging, mainly due to incomplete insights of the regulatory mechanism of CSCs. Here, we unveiled a novel role of ISG15 in the modulation of ATC CSCs.

**Methods:**

The expression of ubiquitin-like proteins were detected by bioinformatics and immunohistochemistry. The correlation between ISG15 expression and tumor stem cells and malignant progression of ATC was analyzed by single-cell RNA sequence from the Gene Expression Omnibus. Flow cytometry combined with immunofluorescence were used to verify the enrichment of ISG15 and ISGyaltion in cancer stem cells. The effect and mechanism of ISG15 and KPNA2 on cancer stem cell-like characteristics of ATC cells were determined by molecular biology experiments. Mass spectrometry combined with immunoprecipitation to screen the substrates of ISG15 and validate its ISGylation modification. Nude mice and zebrafish xenograft models were utilized to demonstrate that ISG15 regulates stem cell characteristics and promotes malignant progression of ATC.

**Results:**

We found that among several ubiquitin proteins, only ISG15 was aberrantly expressed in ATC and enriched in CSCs. Single-cell sequencing analysis revealed that abnormal expression of ISG15 were intensely associated with stemness and malignant cells in ATC. Inhibition of ISG15 expression dramatically attenuated clone and sphere formation of ATC cells, and facilitated its sensitivity to doxorubicin. Notably, overexpression of ISGylation, but not the non-ISGylation mutant, effectively reinforced cancer stem cell-like characteristics. Mechanistically, ISG15 mediated the ISGylation of KPNA2 and impeded its ubiquitination to promote stability, further maintaining cancer stem cell-like characteristics. Finally, depletion of ISG15 inhibited ATC growth and metastasis in xenografted mouse and zebrafish models.

**Conclusion:**

Our studies not only provided new insights into potential intervention strategies targeting ATC CSCs, but also uncovered the novel biological functions and mechanisms of ISG15 and ISGylation for maintaining ATC cancer stem cell-like characteristics.

**Graphical Abstract:**

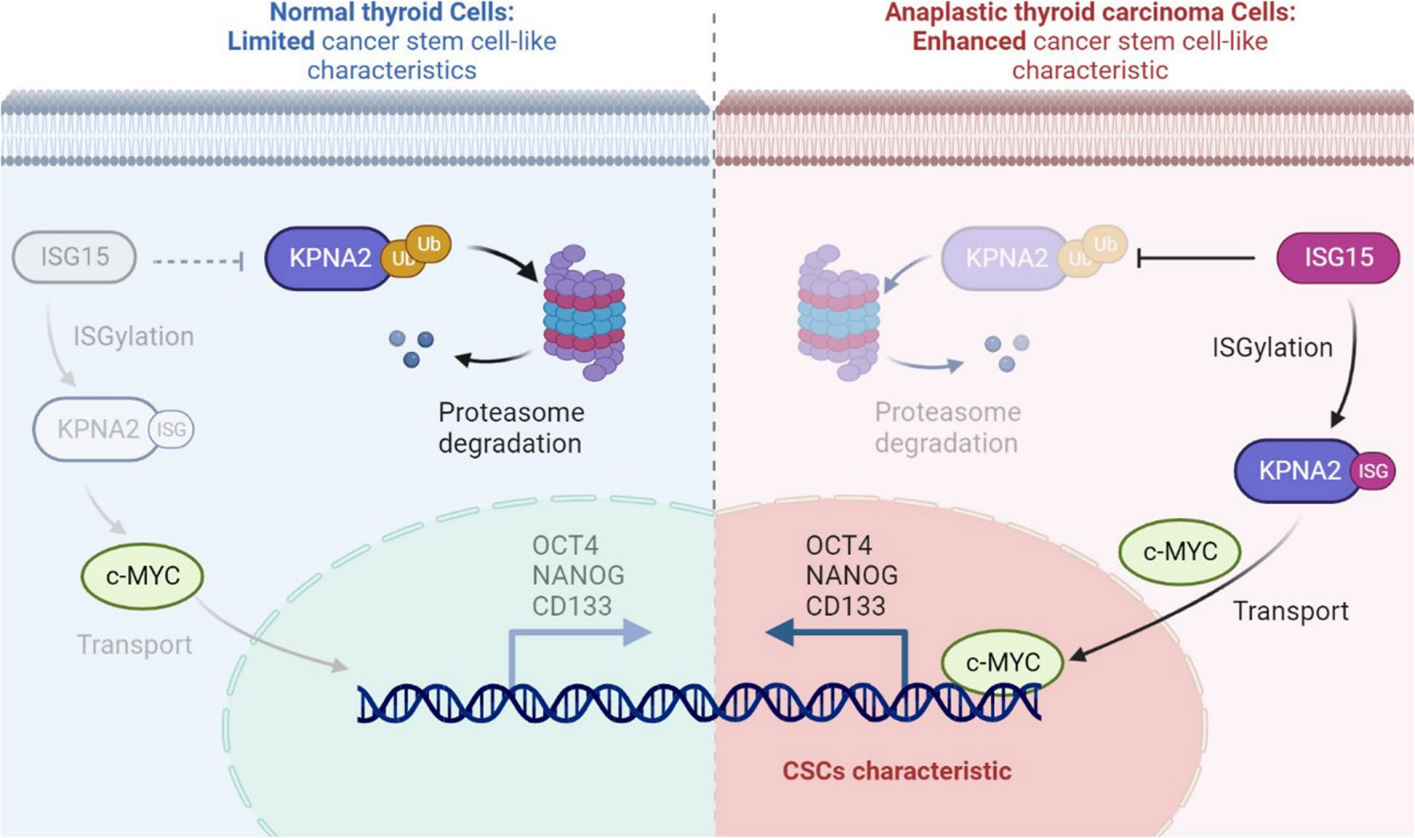

**Supplementary Information:**

The online version contains supplementary material available at 10.1186/s13046-023-02751-9.

## Introduction

Thyroid cancer was one of the fastest growing malignant endocrine tumors in the last 20 years, and there were nearly 586,000 new cases of thyroid cancer worldwide in 2020, posing a serious threat to human health [1, 2]. Anaplastic thyroid carcinoma was a rare subtype of thyroid carcinoma. According to the guidelines of American Joint Committee on Cancer, ATC was usually classified as the stage IV, with a median survival time of less than 6 months and 5-year survival rate of only 8% [3–5]. Due to the extremely rapid progression of ATC, there was no effective treatments to prolong patients’ survival [6–8]. Therefore, there was an urgent need for identifying the pivotal mechanism driving the malignant phenotypes of ATC and finding new therapeutic targets.

Cancer stem-like cells were the crucial cell subset in cancer cells, with self-renewal ability, heterogeneity and high tumorigenicity, mediating malignant proliferation and immune escape of cancers [9–11]. Previous studies had shown that ATC cells were derived from oncogenic mutations in the genome of thyroid stem cells [12], implying that ATC might be a CSCs-driving malignancy. Currently, the main causes of cancer clinical treatment failure were metastasis, recurrence, resistance to chemotherapy and radiotherapy, and avoidance of immunological surveillance, all of which were closely modulated by CSCs [13, 14]. Distinctly, the intervention of CSCs could be considered as the most promising cancer therapeutic strategy.

ISG15, Interferon-stimulated gene 15, was one ubiquitin-like protein that acted an antiviral agent in the immune system by regulating interferon signals or binding viral proteins to prevent its replication [15, 16]. The structure of ubiquitin-like proteins was similar to that of ubiquitin in that amino acid residues with a characteristic sequence at the C-terminal are covalently modified with the substrate protein. So far, there were 8 ubiquitin-like proteins that had been identified and widely recognized as protein modifiers in humans: ISG15, ATG8, FAT10, NEDD8, SUMO1, UFM1, URM1 and ATG12 [17, 18]. Several independent studies had indicated that a novel role for ISG15 in cancer stem cell-like characteristics, regardless of its antiviral effect. For instance, ISG15-mediated tumor stemness was shown to be the important cause of cisplatin resistance in ovarian cancer [19]. And the self-renewal, invasion and tumorigenic potential of CSCs enhanced by ISG15 were crucial for the development of pancreatic cancer [20]. These observations forcefully supported the significance of ISG15 in regulating cancer stem cell-like characteristics, but the mechanisms were muddy. Moreover, it was unclear whether such the stemness regulatory function of ISG15 also exhibited in ATC cells, especially in its cancer stem cells.

In this study, we found that ISG15-mediated ISGylation of KPNA2 inhibited its ubiquitination degradation, thereby promoting nuclear translocation of c-MYC and maintaining cancer stem-like characteristics in ATC.

## Materials and methods

### Microarray information

These datasets GSE76039 (17 poorly-differentiated thyroid carcinomas and 20 ATCs) [21], GSE33630 (45 normal tissues, 49 papillary thyroid carcinomas and 11 ATCs) [22] and GSE29265 (20 normal tissues, 20 papillary thyroid carcinomas and 9 ATCs) [23] were acquired from the Gene Expression Omnibus (https://www.ncbi.nlm.nih.gov/geo/). The batch effects between datasets were corrected by the Remove Batch Effect function of limma package, while retaining the differences between groups in the R4.1.3 environment. And these datasets were performed on the platform of Affymetrix HT HG-U133 + PM Array.

The individual cancer stages and nodal metastasis status analysis of ISG15 in thyroid cancers were applied by the UALCAN (http://ualcan.path.uab.edu/). The mRNAsi was used to evaluate the stem cell characteristics of cancer cells. This mRNAsi was obtained by analyzing and calculating the transcriptome and epigenetic feature sets correlated to stemness by an innovative one-class logistic regression machine-learning algorithm [24]. Subsequently, we analyzed the correlation between ISG15, KPNA2 expression and mRNAsi, and plotted with the ggpubr package. The proteins interacted with ISG15 were predicted via GeneMANIA (https://genemania.org/).

### Single cell RNA-seq (scRNA-seq) analysis

These datasets GSE148673 [25] and GSE29265 containing ATC samples or healthy thyroid samples (NT) were acquired from the Gene Expression Omnibus. The Seurat package was used in R4.1.3 to merge and performed quality control of the scRNA sample data. Cells with the nFeature_RNA below 200 or above 8000 and the percent.mt less than 20% were filtered out. FindVariableFeatures function was used to identify the highly variable genes. FindNeighbors and FindClusters functions were used to perform the cluster analysis. UMAP method was used for dimensionality reduction and visualization. The organized cells were initially annotated into immune, stromal and epithelial cells according to cell markers, and then the epithelial cells were further subdivided. The copy number variation (CNV) score was calculated by the inferCNV package [26] to annotate the benign and malignant cells, in which 1000 normal thyroid cells were randomly selected as the control cells with normal copy number. The cell trajectory was analyzed by the reduceDimension function and DDRTree method of the Monocle package.

### Cell culture

The normal thyroid (NT) cell line Nthy-ori 3 − 1 (NTHY, Fenghui Biotechnology, SC2022010901), PTC cell line BCPAP (Procell, CL-0575), ATC cell lines 8505 C (Fenghui Biotechnology, CL0015) and KHM5M (Procell, CL-0623) were cultured in RPMI-1640 medium supplemented with 10% FBS (NEWZERUM, FBSE500) in atmosphere containing 5% CO2 at 37 °C.

### siRNA transfection and lentiviral transduction

For siRNA transfection, ATC cells were seeded in 6-well plated (Jet Biofil, CAP011006) with 30–40% confluence, and the siRNAs were transfected with jetPRIME (Ployplus, 114 − 15) according to the instruction. The sense sequences of ISG15 siRNA#1 was AUGUCGGUGUCAGAGCUGAA and antisense sequences was UUCAGCUCU- GACACCGACAU. The sense sequences of ISG15 siRNA#2 was UGAGCAUCC- UGGUGAGGAAU and antisense sequences was AUUCCUCACCAGGAUGCUCA. The sense sequences of KPNA2 siRNA#1 was CCUGGACACUUUCUAAUCU and antisense sequences was AGAUUAGAAAGUGUCCAGG. The sense sequences of KPNA2 siRNA#2 was UCACCACCAUGCCAAUUCG and antisense sequences was UGCCUUUGACAAUGUCAUC.

For virus production and lentiviral transduction, the recombinant lentiviruses were produced by co-transfecting 293T cells with an ISG15 shRNA mix, Flag-ISG15-wild type (Flag-ISG15-WT) or Flag-ISG15-AA (156/157-GG to AA, ISGylation non-mutant) expression plasmids with a pRD8.9 packaging plasmid and a pMD.G envelope plasmid. Then, the ATC cells with 30–40% fusion in plates were added with virus accompanied by 5ug/ml polybrene. After 48 h, 5ug/ml puromycin was added to screen and construct stable expression cell lines. And the ISG15 shRNA mix contained two shRNA sequences: #1-CCGGCATGTCGGTGTCAGAGCTGAACTCGAGTTCAGCTCTG-ACACCGACATGTTTTT and #2-CCGGCTGAGCATCCTGGTGAGGAATCTCGA-GATTCCTCACCAGGATGCTCAGTTTTT.

### Cell proliferation clone formation, and sphere formation assay

For cell proliferation assay, 3000 transfected ATC cells were planted in 96-well plates and cultured for 48 h. The cell viability was detected by CCK8 (FDbio, FD3788) according to the instructions. For clone formation assay, 1000 transfected ATC cells were planted in 12-well plates and cultured for 7 days. Then, it was stained with 0.1% crystal violet and photographed. For sphere formation assay, 2000 transfected ATC cells were planted in low-adsorption 6-well plates and cultured with DMEM/F12 medium for 7 to 14 days, which contained 20ng/ml EGF (Peprotech, AF-100-15), 10ng/ml bFGF (Peprotech, 100-18B) and 2% B27 (Thermo, 12,587,010). Then the spheres were collected and photographed, and their size were measured by the Image pro plus software.

### qRT-PCR

The transfected ATC cells were collected and utilized the RNA-Quick Purification Kit (Esunbio, ES-RN001) to extract total RNA, which was then reversely transcribed into cDNA by the Fast All-in-One RT Kit (Esunbio, ES-RT001). The Super SYBR Green qPCR Master Mix (Esunbio, ES-QP002) was utilized for PCR amplification according to the instructions. The sequence of primers were shown in Table 1.

**Table 1:** The sequence of primers

Gene	forward primer	reverse primer
β-ACTIN	CTGGAACGGTGAAGGTGACA	AAGGAACTTCCTTGAACAATGCA
ISG15	ACTCATCTTTGCCAGTACAGG	CAGCTCTGACACCGACATG
ATG8	GAAGGAGAAAAGATCCGGAAG	CAACAGTAAGGTCAGAGGGC
FAT10	TTCTGTCTCTGGTTTCTGGC	TCACGCTGTCATATGGGTTG
NEDD8	ATTACAAGATTCTAGGTGGTT	GAGTGAGAGGATATGTGATG
SUMO	TCCCTGCAGCCGCGGTGT	CAACAGTAAGGTCAGAGGGC
UFM1	GAATAAATCCTGCACAGACTGC	GAAAGGCAATCGTATGTTCCAAG
URM1	ACATCGAGTCACTTTGCCTG	GCATCGTTAATCAGCACCAG
ATG12	TCTTCCGCTGCAGTTTCC	AATGAGTCCTTGGATGGTTCG
OCT4	AATTTGTTCCTGCAGTGCCC	CTCTCGTTGTGCATAGTCGC
NANOG	TGAGTGTGGATCCAGCTTGT	TCTCTGCAGAAGTGGGTTGT
CD133	ACAGCGATCAAGGAGACCAA	GTCAAGTTCTGCATCCACGG

### Western blot and co-immunoprecipitation

For western blot, the collected ATC cells were lysed with RIPA lysis buffer (Applygen, C1053-100). The protein samples were then quantified and denatured, separated by 10% SDS-PAGE gel electrophoresis and transferred to the PVDF membrane (Millipore, IPFL00010). The PVDF membrane was blocked with 5% BSA at room temperature for 1 h, then ISG15 (Proteintech, 15981-1-AP), GAPDH (Abclonal, AC001), CD133 (Proteintech, 66666-1-Ig), Flag (Abclonal, AE005), KPNA2 (Proteintech, 10819-1-AP), or Ub (Abclonal, A2129) antibodies were utilized for the primary antibodies incubating overnight and the corresponding secondary antibodies were incubated at room temperature for 1 h and imaging.

For co-immunoprecipitation, collected ATC cells were lysed by RIPA lysis buffer, and incubated with ISG15 or KPNA2 antibody added to the same amount of total protein extracts at room temperature for 1 h. Then Protein A/G magnetic beads were added and incubated at room temperature for 2 h, washed three times and denatured for western blotting.

### CD133^+^ ATC cell separation and flow cytometry

For CD133^+^ ATC cell separation assay, 1*10^7^ cells were collected and resuspended with 100ul separation buffer. 10ul CD133-PE antibody (Biolegend, S16016B) was added and incubated on ice for 15 min and 10ul PE sorting magnetic beads (Miltenyi, 480,091) were added and incubated on ice for 15 min. Then the mixture was then adsorbed on the magnetic pole for 5 min to remove the supernatant and blow the adsorbed CD133^+^ cells off with PBS.

For flow cytometry, the collected cells were blocked with 2% BSA and incubated at room temperature for 30 min. CD133 or ISG15 antibodies in the ratio of 1:100 were added and incubated at room temperature for 40 min. Then they were incubated with anti-Mouse Alexa Fluor® 488 (Abcam, ab150113) or anti-Rabbit Alexa Fluor® 594 (Abcam, ab150080) for a second time for 30 min, washed twice, and then resuspended with PBS for machine detection. ALDEFLUOR (Stemcell, #01700) assay was performed according to the instructions.

### Immunohistochemistry and immunofluorescence

ATC and NT samples were collected to detect the expression of ISG15. The sample tissues were embedded in paraffin and sliced, then dewaxed and hydrated with xylene and ethanol. These sections were antigenically extracted with 1mM EDTA (pH 8.0) and then blocked with 5% goat serum to reduce nonspecific binding. The primary antibody was cultured with ISG15 (1:200) and the secondary antibody was biotinized for immunohistochemistry.

For immunofluorescence, the treated cells were fixed with paraformaldehyde, blocked and permeated with 2% BSA and 0.2% Triton-X 100. Then the antibodies of CD133 and ISG15 in the ratio of 1:200 were incubated at 4℃ overnight. After 3 times of washing with PBS, the antibodies were added to the secondary fluorescent antibodies and incubated in the dark for 1 h. Finally, after 3 times of PBS cleaning and 5 min of DAPI incubation, imaging was performed.

### Animal models

Firstly, nude mice at 3–4 weeks were randomly divided into two groups, and NC or ISG15-KD 8505 C cells (4*106) were resuspended in PBS containing 10% Matrigel (v/v). Then the suspension was subcutaneously injected into nude mice, and the weight and tumor volume of mice were measured every 2–3 days. Tumor volume = length × width^2^/2. At the end point, mice were euthanized, and tumor tissues were collected and photographed. All animal tumor experiments were approved by the Animal Ethics Committee of Zhejiang Provincial People’s Hospital.

Zebrafish were supplied by Hangzhou Hunter Biotech. Zebrafish were fed in a 28.5 °C recirculating water system (instant sea salt 200 mg/L, electric conductivity: 480 ~ 510µS/cm, pH: 6.9 ~ 7.2) with a standard 12-hour light and dark cycle and fed pellets twice a day. For tumor growth and metastasis models, zebrafish embryos were mechanically delaminated 2 days after fertilization and anesthetized with 0.15 mg/mL tricaine, and each group of 40 zebrafish. 8505 C-NC/ISG15-KD cells were stained with 5 µg/mL DiI (Beyotime, C1036) at 37℃ for 20 min. Then, the cells were cleaned twice and resuspended with PBS, microinjected into the perivitelline space (about 400 cells/piece, 10 µl) with the glass capillary needle and grown at 34 °C in temperature incubator. At the day 3, the zebrafish were anesthetized and then imaged under the fluorescence microscope. Image pro plus software was used to analyze and calculate the optical density of fluorescence signal and metastatic foci to quantitatively evaluate the inhibitory effect of ISG15 knockdown on tumor growth and metastasis in zebrafish. The fluorescence density=(the optical density of fluorescence signal at day 5)/ (the optical density of fluorescence signal at day 2). Schematic diagram was drawn on the Researchers (www.home-for-researchers.com).

### Data analysis

All data were described as mean ± standard deviation of three independent experiments. Statistical significance and differences between two groups were analyzed by unpaired Student’s t-test in GraphPad Prism 7. The asterisk indicated statistical significance (*P < 0.05, **P < 0.01, ***P < 0.001).

## Results

### Ubiquitin-like protein ISG15 was highly expressed in ATC

**Fig. 1:**
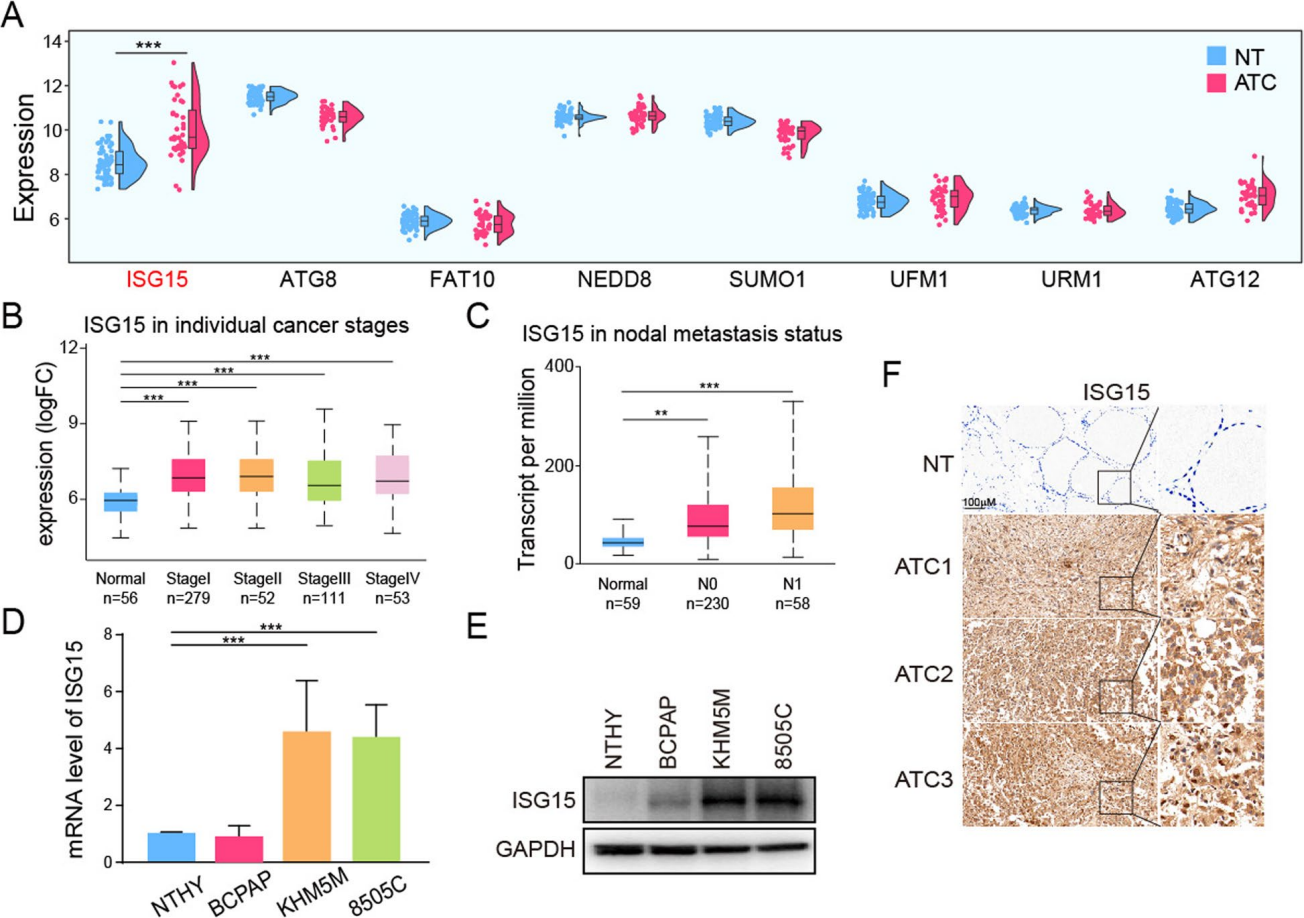
Ubiquitin-like protein ISG15 was highly expressed in ATC. **A** The expression of ubiquitin-like proteins in NT or ATC tissues. **B** The expression of ISG15 in individual thyroid cancer stages. **C** The expression of ISG15 in nodal metastasis status. **D** The mRNA and **E** protein level of ISG15 in different thyroid cancer cell lines. **F** IHC staining to analyze ISG15 expression in clinical ATC patient samples

To explore the role of ubiquitin-like proteins (ISG15, ATG8, FAT10, NEDD8, SUMO1, UFM1, URM1 and ATG12) in thyroid cancer, we integrated the datasets (GSE33630, GSE29265 and GSE76039) and analyzed 65 NT, 69 PTC and 40 ATC samples. The results indicated that only ISG15 was significantly up-regulated in ATC tissues (Fig. 1A). According to the analysis results of THCA samples from the TCGA database, ISG15 was highly expressed in different stages of thyroid cancer, especially ISG15 was positively increased with lymph node metastasis in patients (Fig. 1B, C). Moreover, the results of in vitro experiments showed that ISG15 was increased in ATC compared to NT, and similar trends were observed for its mRNA and protein levels in ATC cell lines (Fig. 1D, E). And as shown in IHC staining in Fig. 1F, the expression of ISG15 in ATC was distinctly higher than that in NT. These results suggested that ISG15 might act as the pivotal factor for driving ATC progression.

### scRNA-seq revealed that abnormal expression of ISG15 were associated with stemness and malignant cells

**Fig. 2:**
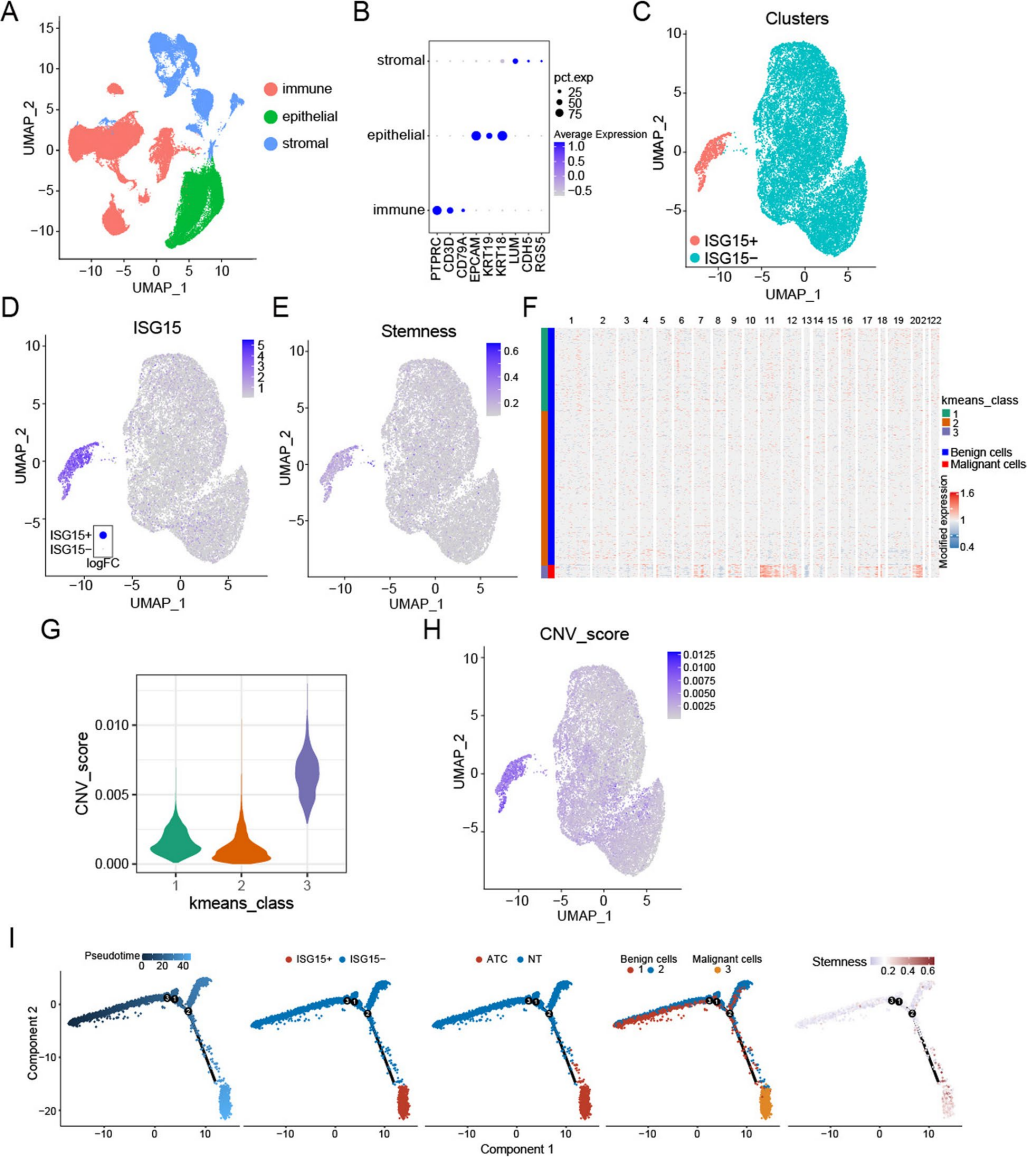
Abnormal expression of ISG15 were associated with stemness and malignant cells. **A** The UMAP plot of 3 cell subsets. **B** The expression of cell markers in different cell subsets. **C** The UMAP plot of ISG15 positive and negative epithelial cell subsets. **D** The expression of ISG15 in different epithelial cell subsets. **E** The stemness scores in the epithelial cells. **F** The CNV scores calculated by the inferCNV in the epithelial cells. **G** The CNV scores of different cell classes grouped by the Kmeans. **H** The UMAP plot of CNV scores in the epithelial cells. **I** The trends of ISG15 expression, malignant cells and stemness along the trajectory of ATC progression

Based on the possibility of ISG15 to dominate cancer stem cell-like characteristics in ATC, two scRNA-seq datasets (GSE148673 and GSE29265) were integrated for analysis. After quality control, a total of 72,708 cells were obtained and annotated into immune (PTPRC, CD3D and CD79A), epithelial (EPCAM, KRT19 and KRT18), and stromal (LUM, CDH5 and RGS5) cell subsets by cell markers (Fig. 2A, B). Next, we subdivided the epithelial cells into different cell subsets for investigating the expression level of ISG15 (Fig. 2C, D). It was obvious that epithelial cells with different expression levels of ISG15 evolved into extremely distinct clusters. Further analysis of the epithelial cells revealed a higher stemness in ISG15-positive cell population than the negative (Fig. 2E).

Then the benign or malignant epithelial cells were evaluated by calculating the CNV score of each cells (Fig. 2F). The epithelial cells were grouped by the unsupervised learning-clustering algorithm K-means, in which class 3 with the sharp rise in CNV scores was identified as malignant cells, while the others were considered as the benign cells (Fig. 2G). Similarly, malignant cell populations with high CNV scores basically overlapped with those with high ISG15 expression and high stemness (Fig. 2H). To investigate the malignant progression of ATC cells, we performed cell trajectory analysis to determine the ISG15 expression, stemness and malignant cells along the trajectory (Fig. 2I). As expected, the number of malignant cells increased sharply during the evolution of NT into ATC, as well as ISG15-positive cells, indicating the correlation of ISG15 expression with ATC malignant cells and its potential association with ATC progression. Intriguingly, the cells localized at the end of the ATC progression route showed higher stemness, further supporting the possibility that ATC was a CSCs-driven malignancy. These data indicated that ISG15 might be critical for the stemness and malignant progression of ATC.

### ISG15 and ISGylation were significantly enriched in cancer stem cells

**Fig. 3:**
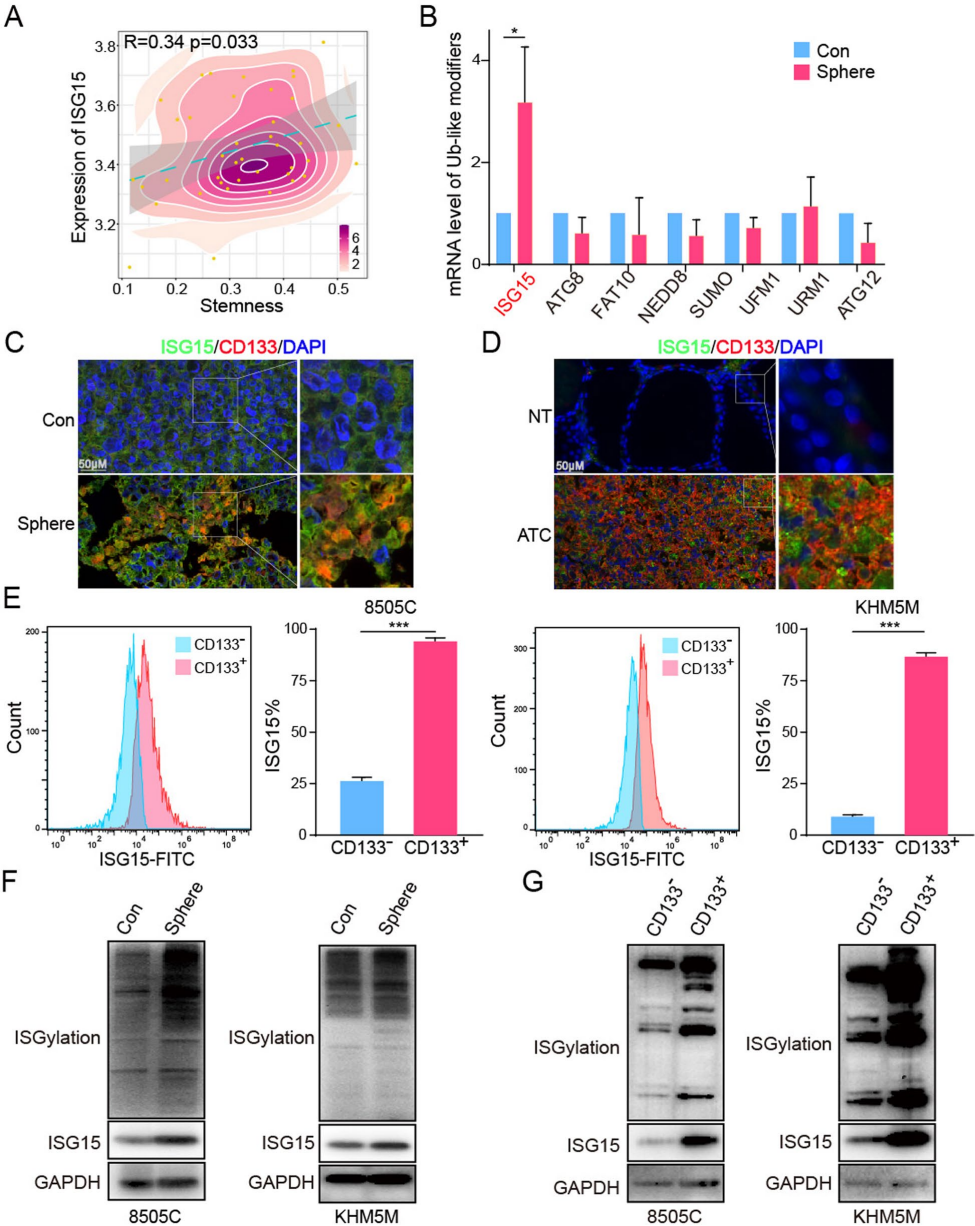
ISG15 and ISGylation were significantly enriched in cancer stem cells. **A** The Pearson correlation analysis of ISG15 and stemness. **B** The mRNA level of ubiquitin-like proteins in con or sphere groups. Immunofluorescence staining of ISG15 and stemness marker CD133 in (**C**) control or sphere groups and (**D**) clinical ATC patient samples. **E** Flow cytometry analysis of ISG15 expression in CD133 + 8505 C or KHM5M cells. Western blot to analyze ISG15 and ISGylation expression in (**F**) control or sphere groups and **G** CD133^±^ ATC cells

In consideration of ATC featured with high cancer stem cell-like characteristics, we tried hard to determine whether high expression of ISG15 was inseparable from cancer stem cells. The correlation analysis of the above integrated datasets showed that the expression of ISG15 was positively correlated with the stemness of ATC, *R* = 0.34 and *P* = 0.033 (Fig. 3A). Meanwhile, we investigated the difference of ubiquitin-like proteins expression in ATC spheres and adhesion cells for validation (Fig. 3B). As expected, ISG15 expression increased rapidly in the ATC spheres compared to other ubiquitin-like proteins. And paraffin sections of ATC spheres combined with immunofluorescence showed that ISG15 and CD133 were upregulated and had stronger co-localization in stem cell spheres (Fig. 3C). A similar trends were observed for its expression and co-localization in ATC clinical paraffin sections (Fig. 3D). Furthermore, we observed by flow cytometry that the expression of ISG15 in CD133^+^ ATC cells (8505 C: 93.7%, KHM5M: 86.5%) was several times higher than that in CD133^−^ (8505 C: 26.1%, KHM5M: 8.8%) (Fig. 3E). Since ISG15 was the crucial modifier of ISGyaltion modification, we examined the level of ISGyaltion in ATC spheres and CD133^+^ cells. The data showed that ISGyaltion was significantly increased in ATC stem cells (Fig. 3F, G).

### ISG15 and ISGylation were critical for maintaining cancer stem cell-like characteristics

**Fig. 4:**
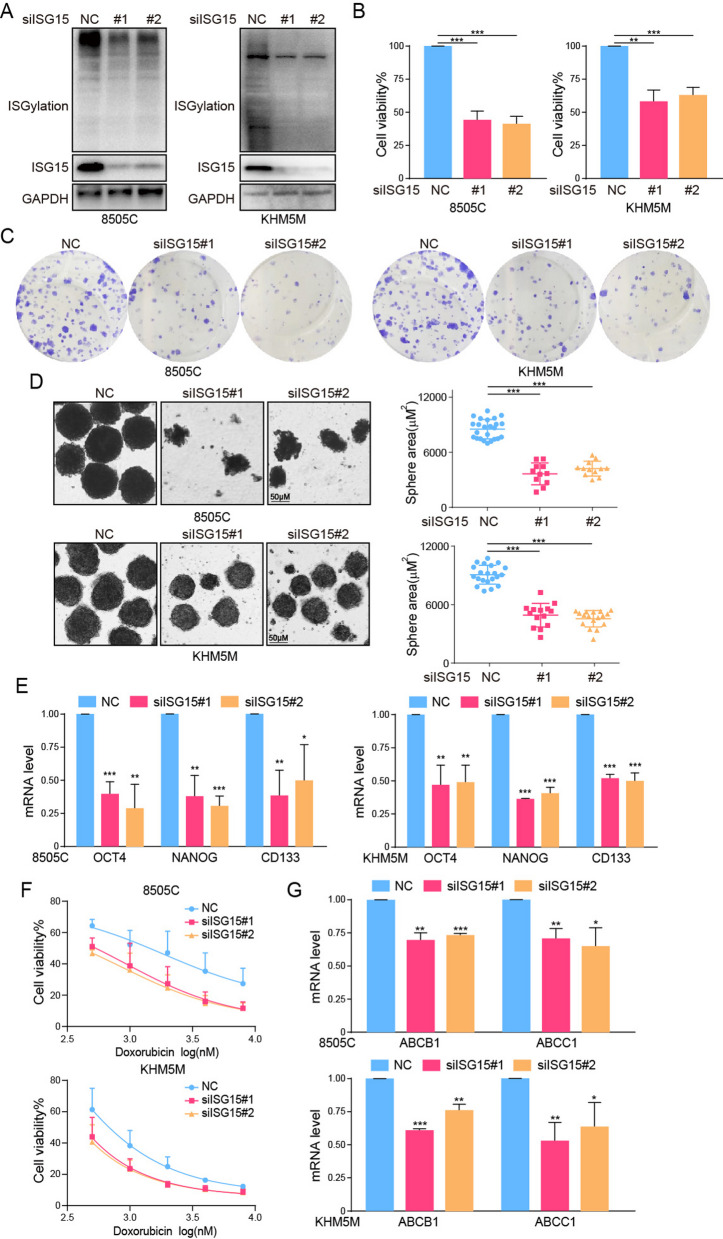
The silence of ISG15 inhibited CSCs characteristics. **A** The knockdown efficiency of ISG15 and ISGylation analyzed by western blot. **B** The cell viability, (**C**) clone formation, (**D**) sphere formation and (**E**) mRNA level of OCT4, NANOG and CD133 of 8505 C or KHM5M cells after ISG15 silence. **F** The cell viability of 8505 C or KHM5M cells after ISG15 silence combined with doxorubicin (0, 0.5, 1, 2, 4 and 8 µM) for 48 h. **G** The mRNA level of ABCB1 and ABCC1 of 8505 C or KHM5M cells after ISG15 silence

Next, we examined whether ISG15 was the critical factor in maintaining cancer stem cell-like characteristics of ATC. ISG15 and ISGylation were inhibited with high efficacy, as confirmed by western blot (Fig. 4A). The deletion of ISG15 obviously influenced the proliferation and cloneformation of ATC cells (Fig. 4B, C). The silence of ISG15 sharply hindered the formation of ATC spheres (Fig. 4D) and impeded the expression of stemness related markers OCT4, NANOG and CD133 (Fig. 4E). Flow cytometry results also indicated that knockdown of ISG15 could impair ALDH activity (Fig. S1A). Moreover, considering that often reduced sensitivity to antitumor drugs was caused by CSCs, we investigated the effect of ISG15 intervention on the sensitivity to doxubicin in ATC. The results showed that the decrease of ISG15 facilitated the sensitivity of ATC cells to doxorubicin and inhibited the expression of classical ATP-binding cassette drug efflux transporters ABCB1 and ABCC1 (Fig. 4F, G).

**Fig. 5:**
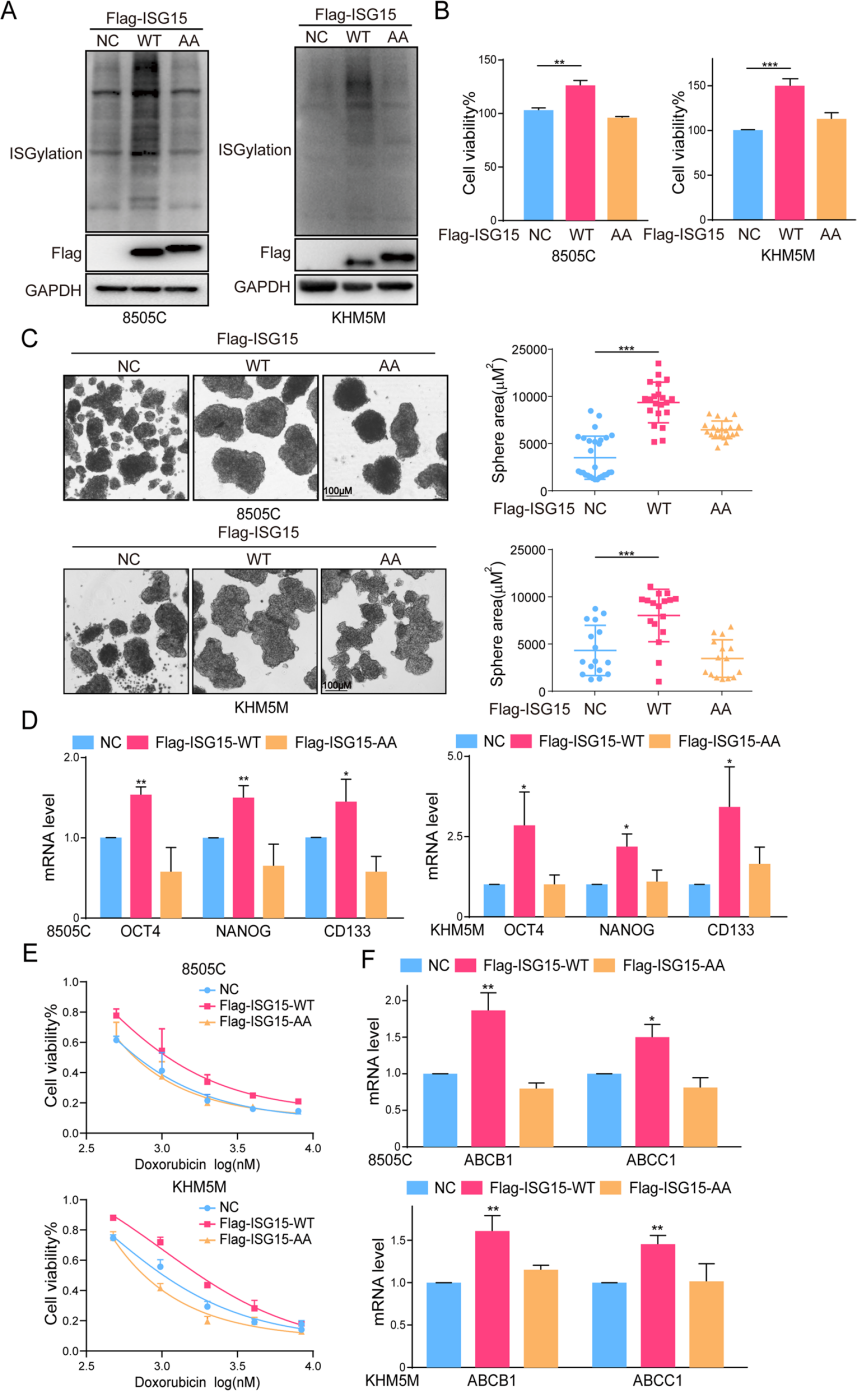
The overexpression of ISGylation promoted CSCs characteristics. **A** The overexpression efficiency of ISGylation-WT/AA analyzed by western blot. **B** The cell viability, (**C**) sphere formation and (**D**) mRNA level of OCT4, NANOG and CD133 of 8505 C or KHM5M cells after ISGylation-WT/AA overexpression. **E** The cell viability of 8505 C or KHM5M cells after ISGylation-WT/AA overexpression combined with doxorubicin for 48 h. **F** The mRNA level of ABCB1 and ABCC1 of 8505 C or KHM5M cells after ISGylation-WT/AA overexpression

We further constructed ISGylation enhanced wild type (WT) and non-modified mutant (AA) cell lines (Fig. 5A). Then, the enhanced ISGylation promoted the proliferation of ATC cells while the non-modified mutations did not (Fig. 5B). Indeed, similar results were found that only the up-regulation of ISGylation promoted the formation of ATC stem cell spheres and increase of OCT4, NANOG and CD133 (Fig. 5C, D). And the increase of ISGylation reduced the sensitivity of ATC cells to doxorubicin and facilitated the expression of ABCB1 and ABCC1 (Fig. 5E, F). Taken together, our results indicated the critical roles of ISG15 and ISGylation in the maintaining cancer stem cell-like characteristics.

### KPNA2 was the pivotal substrate for ISG15 to modulate cancer stem cell-like characteristics in ATC

**Fig. 6:**
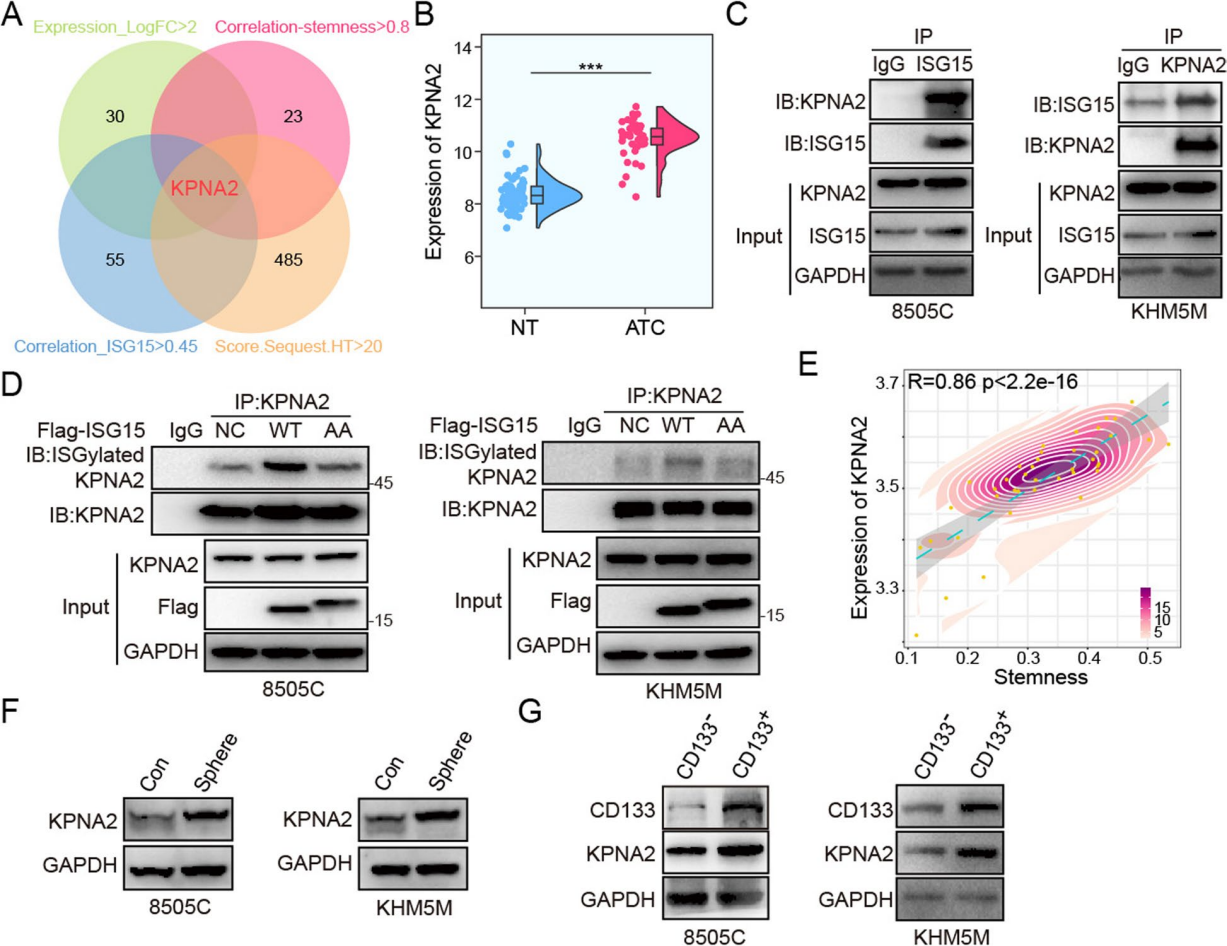
KPNA2 was the substrate for ISG15 to modulate CSCs characteristics. **A** The Venn diagram for screening potential substrates. **B** The expression of KPNA2 in NT or ATC tissues. **C** Co-immunoprecipitation to detect the interaction of endogenous ISG15 with KPNA2 in ATC cell lines. **D** Co-immunoprecipitation to detect the interaction of exogenous ISGylation-WT/AA with KPNA2. **E** The Pearson correlation analysis of KPNA2 and stemness. Western blot to analyze KPNA2 expression in (**F**) control or sphere groups and (**G**) CD133^±^ ATC cells

For delineating the mechanism of ISG15 and ISGylation in modulate cancer stem cell-like characteristics, the physical interaction analysis was conducted to screen a range of potential substrates, including Karyopherin α2 (KPNA2) (Fig. S1B, C). At the same time, the potential proteins binding to ISG15 were enriched and detected by mass spectrometry. Then, the expression, correlation with stemness, correlation with ISG15 expression, and binding score with ISG15 of all potential substrates were intersected (Fig. 6A). Finally, KPNA2 was identified as the candidate substrate for ISG15 to regulate ATC stemness, which was elevated in ATC of above integrated datasets (Fig. 6B).

We subsequently harvested cells for co-immunoprecipitation to explore whether KPNA2 was a ISG15-binding protein. As shown in Fig. 6C, KPNA2 was present in proteins precipitated by ISG15, and vice versa. In addition, the overexpressing ISGyaltion-WT/AA cells were employed to determine the ISGylation of KPNA2. The results of co-immunoprecipitation showed that ISGylation of KPNA2 was enhanced after overexpression of Flag-ISG15-WT, while Flag-ISG15-AA did not (Fig. 6D). More importantly, the expression of KPNA2 highly correlated with the stemness of ATC samples, and negatively correlated with recurrence free survival of ATC patients (Fig. 6E and Fig. S1D). And compared to control or CD133^−^ ATC cells, CD133^+^ ATC cells or spheres had markedly up-regulated KPNA2 (Fig. 6F, G). Thus, KPNA2 might be involved in the regulation of ATC stemness and the pivotal potential substrate for ISG15 and ISGylation.

### Deletion of ISGylation promoted the ubiquitination and degradation of KPNA2

**Fig. 7:**
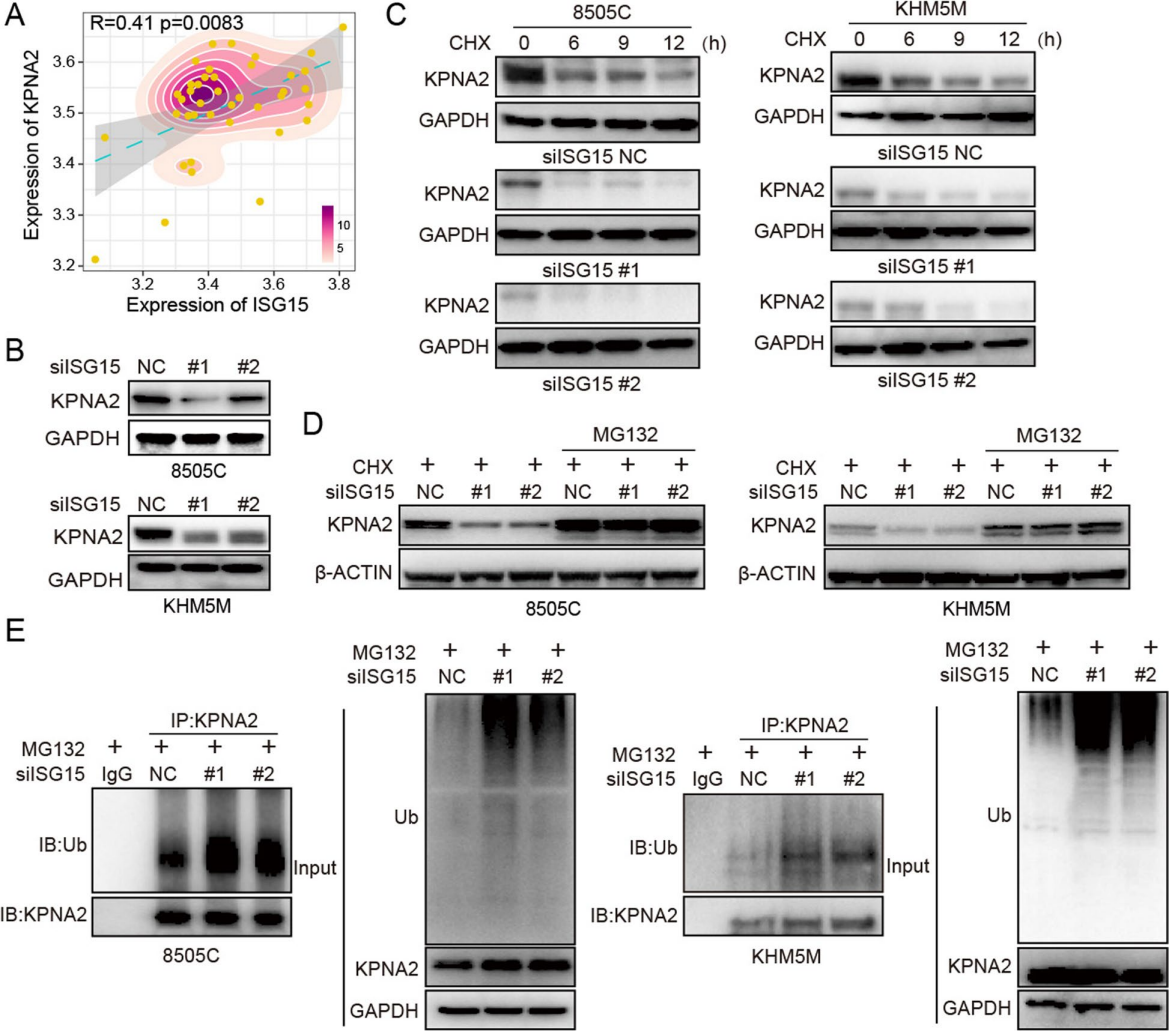
Deletion of ISGylation promoted the ubiquitination and degradation of KPNA2. **A** The pearson correlation analysis of KPNA2 and ISG15. **B** Western blot to analyze KPNA2 expression after ISG15 silence in ATC cells. **C** Western blot to analyze KPNA2 expression after ISG15 silence combined with 20 ug/ml Cycloheximide (CHX) at the 0, 6, 9 and 12 h. **D** Western blot to analyze KPNA2 expression after ISG15 silence combined with 10 mM MG132 or not. **E** Co-immunoprecipitation to detect the ubiquitination of KPNA2 after ISG15 silence combined with 10 mM MG132

Since KPNA2 could be modified by ISG15 directly and correlated with its protein levels, we hypothesized that ISG15 exerted such regulation through the ubiquitination of KPNA2. In order to better illustrate the relationship between KPNA2 and ISG15, correlation analysis in above integrated datasets was utilized and found that the expression of KPNA2 was positively correlated with ISG15 (Fig. 7A). Based on the correlation, we investigated the expression level of KPNA2 after silencing ISG15. As showed in Fig. 7B, the protein level of KPNA2 was significantly inhibited by the reduction of ISG15, thus raising the possibility that the proteolytic ubiquitination. And the protein stability of KPNA2 was significantly decreased after inhibition of ISG15 (Fig. 7C). Next, the proteasome inhibitor MG132 was performed to detect the association between down-regulation of KPNA2 and ubiquitin-proteasome degradation (Fig. 7D). The degradation of KPNA2 induced by ISG15-silence could be rescued by MG132. Furthermore, the effects of ISG15 and ISGyaltion on the ubiquitination level of KPNA2 were investigated by co-immunoprecipitation combined with MG132. Notably, ubiquitination of KPNA2 was sharply enhanced after the inhibition of ISG15 and ISGylation (Fig. 7E). Therefore, these results collectively revealed the specific regulation of ubiquitination degradation of KPNA2 by ISG15 and ISGylation.

### KPNA2 mediated cancer stem-like characteristics of ATC regulated by ISG15 and ISGylation

**Fig. 8:**
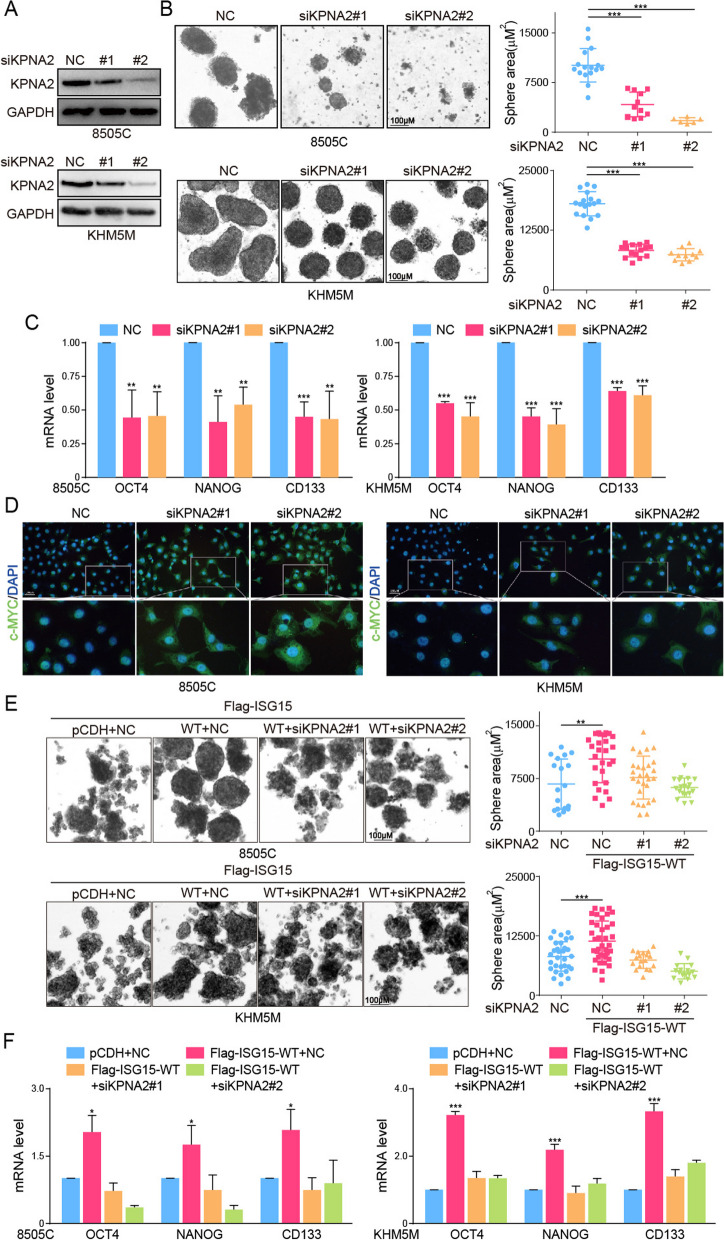
KPNA2 mediated CSCs characteristics regulated by ISG15 and ISGylation. **A** The knockdown efficiency of KPNA2 analyzed by western blot. **B** The sphere formation and (**C**) mRNA level of OCT4, NANOG and CD133 of ATC cells after KPNA2 silence. **D** Immunofluorescence staining of c-MYC after KPNA2 silence in ATC cells. **E** The sphere formation and (**F**) mRNA level of OCT4, NANOG and CD133 after ISGylation-WT overexpression combined with KPNA2 silence or not

Aforementioned data suggested that KPNA2 was precisely modulated by ISG15 and strongly correlated with ATC stemness. In this context, we examined the effects of KPNA2 on cancer stem-like characteristics of ATC cells. The formation of ATC spheres was hindered obviously by the silence of KPNA2 (Fig. 8A, B). Moreover, this deletion of KPNA2 sharply impeded the expression of OCT4, NANOG and CD133 (Fig. 8C), as well as inhibiting the ALDH activity (Fig. S1E). KPNA2, a member of the nuclear transporter family, had recently been found to be involved in the nuclear entry of cancer stemness-associated transcription factor c-MYC [27, 28]. Based on the ATC datasets, c-MYC was found to be significantly up-regulated in ATC and positively correlated with the stemness index of ATC samples (Fig. S1F, G). To expand our exploration of the mechanism that KPNA2 mediated ATC stemness regulated by ISG15, we evaluated whether KPNA2 promoted nuclear localization of c-MYC in ATC cells (Fig. 8D). By immunofluorescence staining, we found that higher levels of c-MYC were blocked in cytoplasm by inhibition of KPNA2. Subsequently, we examined the roles of KPNA2 in ISG15 promoting the stemness of ATC cells. ISG15 was overexpressed in ATC cells, and then KPNA2 was silenced on this basis. Our results indicated that the down-regulation of KPNA2 could inhibit the formation of ISG15-promoted ATC sphere and the expression of OCT4, NANOG and CD133 (Fig. 8E, F). Taken together, these data demonstrated that KPNA2 mediated cancer stem-like characteristics of ATC reinforced by ISG15 and ISGylation.

### ISG15 was indispensable for the tumorigenesis of ATC cells in vivo

**Fig. 9:**
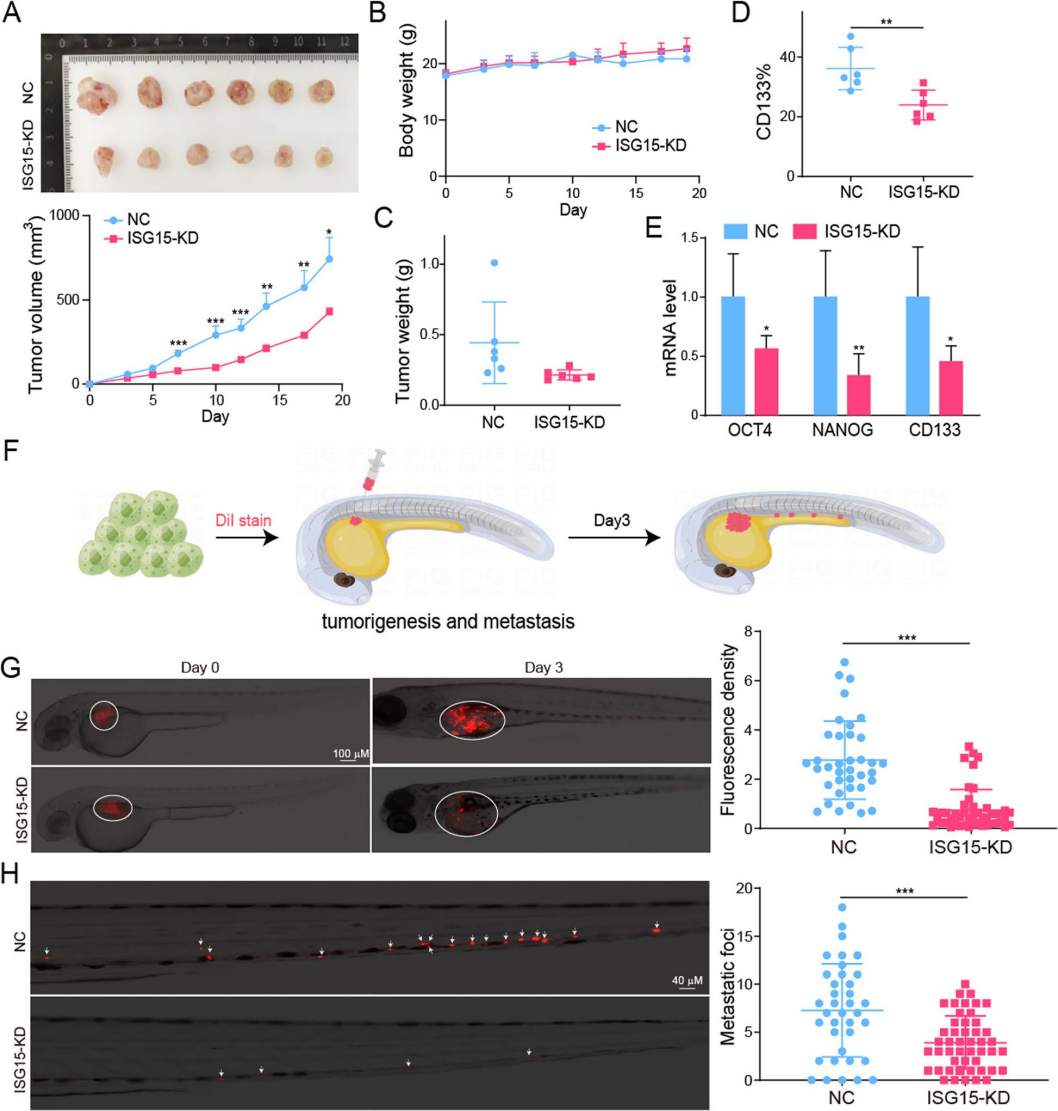
Knockdown of ISG15 inhibited tumorigenesis and metastasis of ATC in vivo. Subcutaneous tumor xenografts in nude mice established by 8505 C-NC or ISG15-KD. **A** The growth curve of the tumor. **B** mice body weight. **C** Tumor weight. **D** Flow cytometry analysis of CD133 expression in tumor tissues. **E** The expression of ISG15, ISGylation, KPNA2 and stemness markers analyzed by PCR. **F** Schematic diagram of the zebrafish models. **G** The tumorigenesis of zebrafish in control and ISG15-KD groups. **H** The metastasis of zebrafish in control and ISG15-KD groups. Red fluorescence represents labeled ATC tumor cells

To determine the effect of ISG15 intervention on tumor growth in vivo, control or ISG15-knockdown cells were established and subsequently injected subcutaneously into nude mice. Although animals in both groups showed analogous body weights, the ISG15-knockdown mice indicated obviously lower rates of tumor volume, which associated with lower levels of ISG15 (Fig. 9A, B). At the end of the experiments, the weight of tumor tissues in ISG15-knockdown mice was significantly less than that in control group (Fig. 9C). Flow cytometry detected a decrease of CD133 expression in the ISG15-knockdown mice (Fig. 9D). And down-regulated stemness related markers were showed in ISG15 knockout xenografts (Fig. 9E).

Simultaneously, we also confirmed the inhibitory effect of ISG15 deletion on ATC tumorigenesis and metastasis in zebrafish models. First, we successfully constructed the zebrafish xenograft tumor model with ATC cells. 8505 C was labeled with fluorescence, and then microinjected into the perivitelline space of Zebrafish. After 3 days, the fluorescence signal at the injection site was significantly enhanced and foci of tumor cell metastasis in the tail vein was observed (Fig. 9F). Obviously, ISG15 knockdown greatly decreased the mean fluorescence intensity of ATC xenograft from 2.77 to 0.74 and the mean number of metastatic foci from 7.27 to 3.90 (Fig. 9G, H). Collectively, these results are consistent with the notion that inhibition of ISG15 lead to stemness block and impeded their malignant phenotypes in ATC cell lines, further supporting that intervention of ISG15 and ISGylation may be a promising treatment strategy for ATC.

## Discussion

ATC was characterized by strong aggressivity, high metastasis and lethality. There were more abundant CSCs in ATC than PTC or normal thyroid, which was the core driving force for its fatal and poor prognosis. We also found a large number of CD133-positive cells in ATC cell lines (Fig. S1H). Recent studies had found that about 60% of CD133^+^ CSCs were existed in ATC cells, and the isolated CD133^+^ CSCs exhibited higher radiation resistance and expression of stemness markers, as well as stronger tumorigenicity and metastasis ability [29, 30]. And cisplatin and doxorubicin induced ATC cell death were synergistically promoted by silencing ATC stem cell markers [31]; Intervention of JAK/STAT3 and NF-κB signals in ATC CSCs could effectively impede tumor growth in vivo [32]. In this study, we confirmed that CSCs are highly enriched in ATCs and may be the pivotal maintainer of the malignant phenotypes of ATC. And the depletion of ubiquitin-like protein ISG15 could relieve the malignant growth of ATC, which was a potential therapeutic target for ATC.

The formation and quantitative homeostasis of CSCs were regulated by complex and strict mechanisms, and ubiquitin-like modifiers exhibited essential roles in the homeostasis of CSCs [33, 34]. Recently, the behavior of ubiquitin-like proteins and their modifications in CSCs had attracted increasing attention. It was found that increased SUMOylation significantly promoted the self-renewal and chemotherapy resistance of CSCs in colon cancer [35]. And Neddylation could regulate the proliferation and differentiation of CSCs in lung cancer and colon cancer [36]. However, the roles of ubiquitin-like proteins and their modifications in maintaining the stemness of ATC was poorly understood. Based on the comprehensive analysis of scRNA-seq, we found that ISG15 signal was aggregated in ATC malignant cells accompanied by increased stemness, revealing the inextricable association between the high of ISG15 expression and stemness, which was crucial for the occurrence and development of ATC. Expectedly, our data demonstrate that ISG15 and ISGylation were intensely upregulated in ATC CSCs compared to other ubiquitin-like proteins. Such an increase in ISG15 has been previously reported in other tumor CSCs [19, 37, 38]. We found that inhibition of ISG15 blocked the cancer stem-like characteristics of ATC cells by experiments indeed, and significantly impeded ATC growth and metastasis in xenografted mouse and zebrafish models. Further through overexpression of ISGylation and non-modification mutant, it was found that ISGylation was the core element for ISG15 mediated regulation of cancer stem-like characteristics. However, the mechanisms by which ISG15 and ISGyaltion affected CSCs in these processes remains unexplored.

We analyzed and confirmed the binding of KPNA2 to ISG15 and its ISGylation by mass spectrometry and experiments, and further identified KPNA2 as the mediator of ISG15 in modulating the cancer stem-like characteristics of ATC. KPNA2, a member of the Karyopherin α family that consisted of a cluster of basic amino acids, could function as the linking molecule to transport the carried protein cargoes from the cytoplasm to the nucleus [39, 40]. In addition, the central roles of KPNA1 ~ 7 in the malignant progression of other cancers were increasingly being recognized [41–43]. And Fang et al. found that KPNA4 enhanced nuclear p65 expression and NF-κB activity, and significantly augmented the proliferation and invasion of PTC cells [44]. Notably, only KPNA2 had been found in our previous study to be the crucial transporter of transcription factor CREB3L1 into the nucleus and remodel the tumor microenvironment to promote tumor growth and metastasis in ATC. However, the roles of KPNA2 in the CSCs of ATC was not yet fully characterized [45]. In this study, we demonstrate that KPNA2 silencing effectively blocked CSCs and that its presence was also required for increased ISGyaltion to promote ATC cancer stem cell-like characteristics.

Due to the observation that KPNA2 mediated the modulation of ISGylation to cancer stem cell-like characteristics, the specific mechanisms by which the ISGylation of KPNA2 impacted upon itself aroused our great interest. ISGylation was a ubiquitin-like modification like with ubiquitylation that occurred at lysine residues of proteins, so there was an crucial connection between their functions [46]. Our data indicated that deletion of the ISGylation of KPNA2 reduced its protein levels, implying the roles of dynamic balance of ISGyaltion and ubiquitination. ISGylation could inhibit ubiquitination by blocking connections between ubiquitin chains and target proteins, thereby preventing target proteins from the degradation [47, 48]. For example, ISGylation could regulate the stability of KRAS or β-catenin and maintain the malignant phenotypes of breast cancer and colorectal cancer [48, 49]. In this study, we found that the stability of KPNA2 was controlled by ISGylation and deletion of ISGylation significantly enhanced ubiquitination of KPNA2. This suggested that there was a homeostasis between the ISGylation and ubiquitination of KPNA2 that regulated its expression. We conjectured that ISGylation may either competitively bind to ubiquitination sites or block adjacent ubiquitination sites, and these assumptions needed to be further explored in subsequent researches. It was worth considering to screen specific small molecules targeting key E1 ligases UBE1L, E2 ligase UBCH8 or E3 ligase HERC5 in subsequent studies to down-regulate ISGylation for ATC treatment. And ISGylation inhibitors may be a good choice in combination with other chemotherapy or immune checkpoint inhibitors.

In summary, we have demonstrated for the first time that ISG15 maintains cancer stem cell-like characteristics by inhibiting KPNA2 degradation through ISGylation in ATC. Based on these newly achieved mechanistic understandings, our study not only provides new insights into CSCs driving malignant progression of ATC, but also directs towards the design of new therapies targeting ATC CSCs.

## Supplementary Information

**Additional file 1: Fig. S1.** ISG15 and KPNA2 inhibited CSCs characteristics. (A) Flow cytometry analysis of ALDH activity after ISG15 silence in ATC cells. (B) The GeneMANIA database to analyze the physical interaction of ISG15. (C) The secondary mass spectrometry of KPNA2 interacted with ISG15. (D) Recurrence free survival (RFS) analysis of KPNA2 in thyroid cancer was acquired from the Kaplan–Meier plot database. (E) Flow cytometry analysis of ALDH activity after KPNA2 silence in ATC cells. (F) The expression of c-MYC in NT or ATC tissues. (G) The Pearson correlation analysis of c-MYC and stemness. (H) Flow cytometry analysis of CD133^+^ cell ratio in ATC cells

## Data Availability

The data supporting the conclusions of this paper have been provided in this paper and GEO database. In addition, all data for this study are available from the corresponding author upon reasonable request.
