# Correction: ISG15 and ISGylation modulates cancer stem cell-like characteristics in promoting tumor growth of anaplastic thyroid carcinoma

**DOI:** 10.1186/s13046-024-03226-1

**Published:** 2024-11-13

**Authors:** Tong Xu, Chaozhuang Zhu, Jinming Chen, Feifeng Song, Xinxin Ren, Shanshan Wang, Xiaofen Yi, Yiwen Zhang, Wanli Zhang, Qing Hu, Hui Qin, Yujia Liu, Song Zhang, Zhuo Tan, Zongfu Pan, Ping Huang, Minghua Ge

**Affiliations:** 1 Center for Clinical Pharmacy, Cancer Center, Department of Pharmacy, Zhejiang Provincial People’s Hospital (Afliated People’s Hospital), Hangzhou Medical College, Hangzhou, Zhejiang China; 2 Zhejiang University of Technology, Hangzhou, Zhejiang China; 3 Hangzhou Medical College, Hangzhou, Zhejiang China; 4 Key Laboratory of Endocrine Gland Diseases of Zhejiang Province, Zhejiang Provincial People’s Hospital (Afliated People’s Hospital), Hangzhou Medical College, Hangzhou, Zhejiang China; 5 Otolaryngology & Head and Neck Center, Cancer Center, Department of Head and Neck Surgery, Afliated People’s Hospital, Zhejiang Provincial People’s Hospital, Hangzhou Medical College, Hangzhou, Zhejiang China

**Correction: J Exp Clin Cancer Res 42, 182 (2023)**

**https://doi.org/10.1186/s13046-023-02751-9**

Following the publication of the original article [1], the author identified an error in Fig. 5, specifically, Fig. 5A. The GAPDH strip was misuse and is the same with Fig. 8A.

The correct figure is presented below:

**Incorrect Fig. 5**

**Fig. 5:**
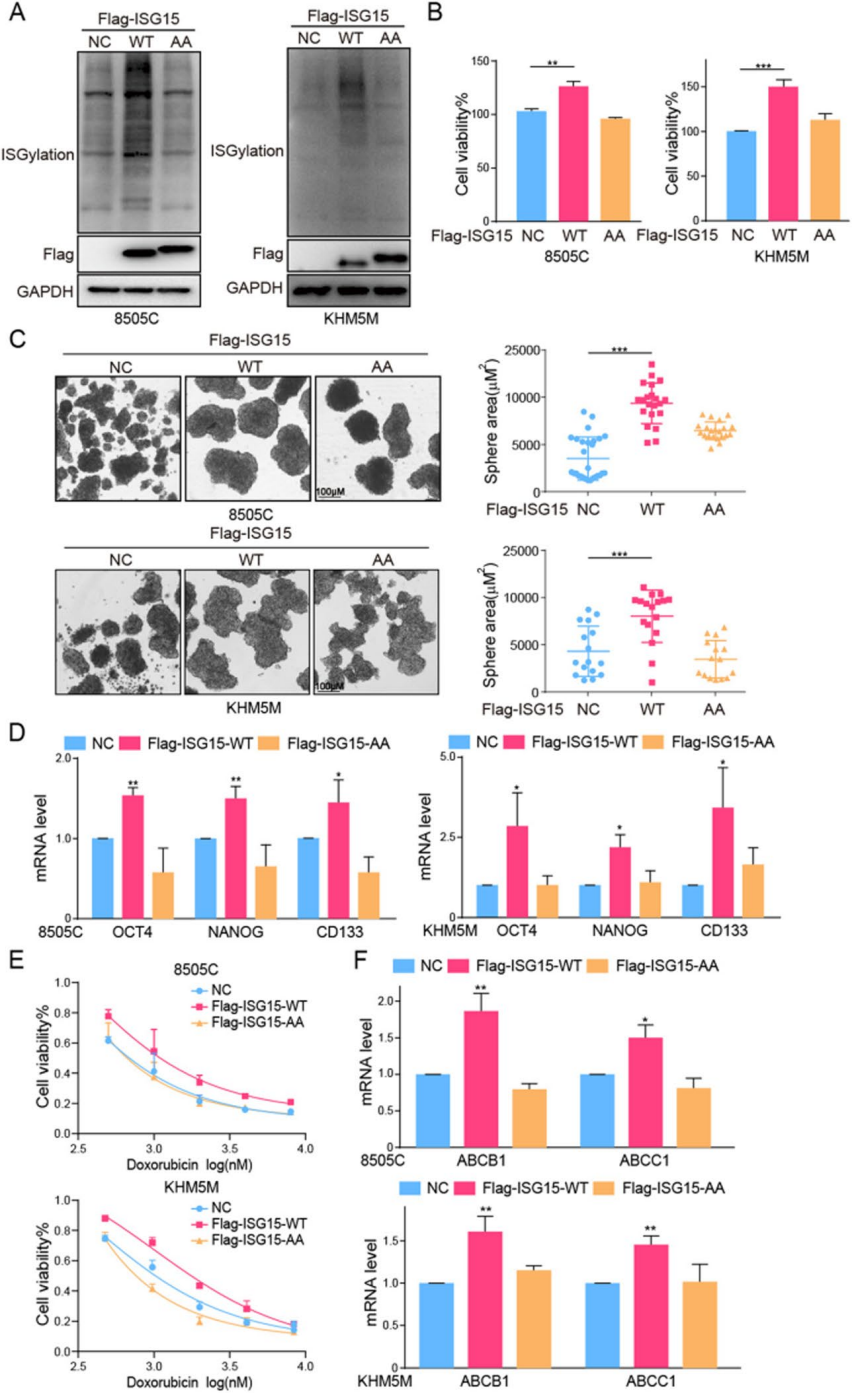
The overexpression of ISGylation promoted CSCs characteristics. **A** The overexpression efciency of ISGylation-WT/AA analyzed by western blot. **B** The cell viability, (**C**) sphere formation and (**D**) mRNA level of OCT4, NANOG and CD133 of 8505 C or KHM5M cells after ISGylation-WT/AA overexpression. **E** The cell viability of 8505 C or KHM5M cells after ISGylation-WT/AA overexpression combined with doxorubicin for 48 h. **F** The mRNA level of ABCB1 and ABCC1 of 8505 C or KHM5M cells after ISGylation-WT/AA overexpression

**Correct Fig. 5**

**Fig. 5:**
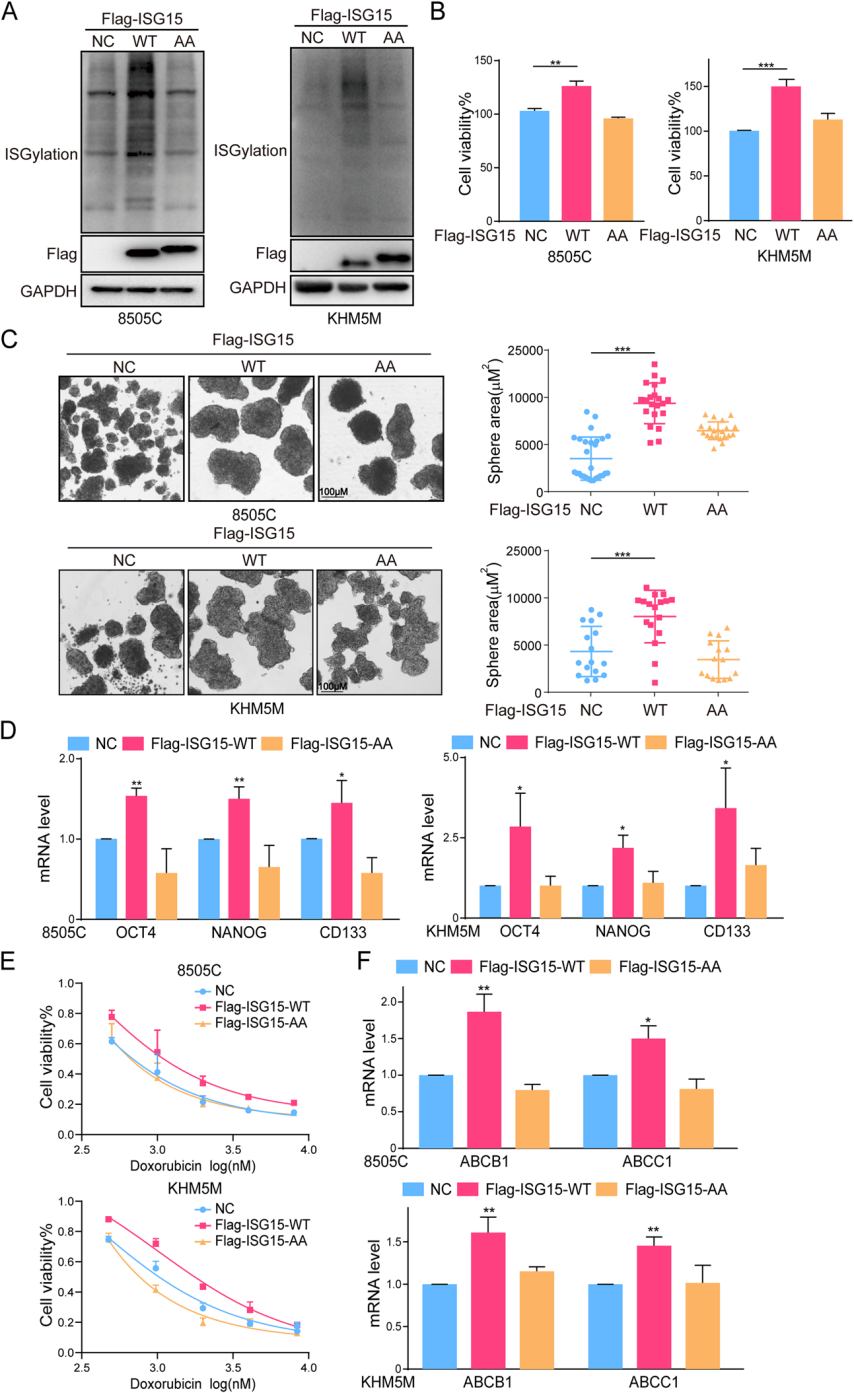
The overexpression of ISGylation promoted CSCs characteristics. **A** The overexpression efciency of ISGylation-WT/AA analyzed by western blot. **B** The cell viability, (**C**) sphere formation and (**D**) mRNA level of OCT4, NANOG and CD133 of 8505 C or KHM5M cells after ISGylation-WT/AA overexpression. **E** The cell viability of 8505 C or KHM5M cells after ISGylation-WT/AA overexpression combined with doxorubicin for 48 h. **F** The mRNA level of ABCB1 and ABCC1 of 8505 C or KHM5M cells after ISGylation-WT/AA overexpression

The correction does not compromise the validity of the conclusions and the overall content of the article. The original article [1] has been updated.
