# Supplementary material for: ISG15 and ISGylation modulates cancer stem cell-like characteristics in promoting tumor growth of anaplastic thyroid carcinoma
Source: J Exp Clin Cancer Res. 2023 Jul 27;42:182. doi: 10.1186/s13046-023-02751-9 (PMC10373324; doi:10.1186/s13046-023-02751-9)
Supplement: Supplementary file 1 — Additional file 1: Fig. S1. ISG15 and KPNA2 inhibited CSCs characteristics. (A) Flow cytometry analysis of ALDH activity after ISG15 silence in ATC cells. (B) The GeneMANIA database to analyze the physical interaction of ISG15. (C) The secondary mass spectrometry of KPNA2 interacted with ISG15. (D) Recurrence free survival (RFS) analysis of KPNA2 in thyroid cancer was acquired from the Kaplan–Meier plot database. (E) Flow cytometry analysis of ALDH activity after KPNA2 silence in ATC cells. (F) The expression of c-MYC in NT or ATC tissues. (G) The Pearson correlation analysis of c-MYC and stemness. (H) Flow cytometry analysis of CD133+ cell ratio in ATC cells [file 13046_2023_2751_MOESM1_ESM.zip › Additional file 1.doc]

**Fig.S1 ISG15 and KPNA2 inhibited CSCs characteristics.** (A) Flow cytometry analysis of ALDH activity after ISG15 silence in ATC cells. (B) The GeneMANIA database to analyze the physical interaction of ISG15. (C) The secondary mass spectrometry of KPNA2 interacted with ISG15. (D) Recurrence free survival (RFS) analysis of KPNA2 in thyroid cancer was acquired from the Kaplan–Meier plot database. (E) Flow cytometry analysis of ALDH activity after KPNA2 silence in ATC cells. (F) The expression of c-MYC in NT or ATC tissues. (G) The Pearson correlation analysis of c-MYC and stemness. (H) Flow cytometry analysis of CD133^+^ cell ratio in ATC cells.
